# Daytime noninvasive ventilatory support for patients with ventilatory pump failure: a narrative review

**DOI:** 10.1186/s40248-019-0202-7

**Published:** 2019-11-30

**Authors:** Paolo Banfi, Paola Pierucci, Eleonora Volpato, Antonello Nicolini, Agata Lax, Dominique Robert, John Bach

**Affiliations:** 1 IRCCS Fondazione Don Carlo Gnocchi, via Capecelatro, 66 20148 Milan, Italy; 2 Cardio Thoracic Department, Respiratory and Sleep Disorders Unit, Bari Policlinic, Bari, Italy; 3 Department of Psychology, Università Cattolica del Sacro Cuore, Milan, Italy; 4 Respiratory Rehabilitation Unit, ASL 4 Chiavarese, Hospital of Sestri Levante, Sestri Levante, Italy; 5 Hospices Civils de Lyon, Hôpital Edouard Herriot, Service de Réanimation Médicale, Lyon, France; 6 Université de Lyon, Université Claude Bernard Lyon 1, Lyon, France; 7 Department of Physical Medicine and Rehabilitation, Rutgers University New Jersey Medical School, Newark, USA

**Keywords:** Neuromuscular disorders, Respiratory failure, Noninvasive ventilation/ventilatory support, Mouthpiece ventilation, Intermittent abdominal pressure ventilation

## Abstract

Over the past three decades, the use of noninvasive ventilation or “NIV” to assuage symptoms of hypoventilation for patients with early onset or mild ventilatory pump failure has been extended to up to the use of continuous noninvasive ventilatory support (CNVS) at full ventilatory support settings as a definitive alternative to tracheostomy mechanical ventilation. NVS, along with mechanical insufflation-exsufflation, now provides a noninvasive option for the management of both chronic and acute respiratory failure for these patients. The most common diagnoses for which these methods are useful include chest wall deformities, neuromuscular diseases, morbid obesity, high level spinal cord injury and idiopathic, primary or secondary disorders of the ventilatory control. Thus, NVS is being used in diverse settings: critical care units, medical wards, at home, and in extended care. The aim of this review is to examine the techniques used for daytime support.

## Introduction

Respiratory muscle insufficiency/failure is defined by the inability to maintain adequate blood oxygenation and carbon dioxide removal [1, 2]. The underlying pathophysiologic processes causing it are quite different from those typically treated by physicians. Instead of lung/airways disorders that result primarily in oxygenation impairment and air trapping, for patients with ventilatory pump failure hypoxia is secondary to inspiratory and/or expiratory muscle impairment that results in hypoventilation and airway mucus congestion [3, 4]. When patients’ respiratory muscles cannot sustain autonomous respiration, the use of mechanical ventilation has become [5] the cornerstone of modern pulmonary medicine and critical care.

Non-invasive ventilation or “NIV” has become synonymous with continuous positive airway pressure and low span bi-level positive airway pressure to treat sleep-disordered breathing (central and obstructive apneas) and acute respiratory failure for patients with lung disease [5–11]. Observational and randomized controlled studies now strongly support NIV for patients with mild neuromuscular disease (NMD) who are not completely respirator dependent [12–19]. Furthermore, when considering NIV, for patients with lung diseases, airway clearance therapy is addressed [20–22]. Standardized guidelines have been accepted worldwide for the initiation of nocturnal NIV [23–32]. However, for patients with ventilatory pump failure advancing disease can result in patients extending sleep NIV into daytime hours and becoming hypercapnic because of the inadequacy of the pressure support settings. These patients can also require mechanical in-exsufflation to increase cough flows [20–22]. The paucity of literature focused on this topic and failure to recognize the deficiencies in the administration of NIV motivated this review, which aims to highlight means for providing up to continuous noninvasive ventilatory support (CNVS).

There is NVS and invasive mechanical ventilation whether via translaryngeal or tracheostomy tube (TMV) [5–8, 33–36]. However, 80% of mortality in some diagnoses of patients using tracheostomy ventilation has been reported to be due to the tube itself, rather than directly to the disease [37]. On the other hand, while tracheostomy ventilation is associated with increased mortality and diminished quality of life [38], CNVS is permitting patients with no autonomous breathing ability and little to no vital capacity (VC) with diagnoses including spinal muscular atrophy type 1 more than 2 decades [39], post-polio survivors to survive for over 65 years [40], Duchenne muscular dystrophy to survive into their 50s on CNVS for over 25 years [41], etc.. Patients who require daytime NVS can eventually require it around-the-clock with little to no autonomous ability to breathe. For this, full respiratory support settings are necessary and “NIV” or typical bi-level positive airway pressure is inadequate. Thus, CNVS refers to the administration of full ventilatory support settings day and night [9, 13]. Its benefits include improving gas exchange, symptoms [42], quality of life [43–45], decreasing the incidence of pneumonia [46], facilitating ventilator weaning, resting respiratory muscles, and supporting life for people indefinitely without need to resort to tracheotomy [46]. Tracheostomy tubes, on the other hand, tend to increase ventilator dependence, reduce quality of life, and are associated with reactive depression [47–50].

A literature review and manual selection using PubMed/Medline was undertaken through May 2019. Search terms used were noninvasive ventilation, noninvasive ventilatory support, home ventilation, diurnal or daytime non-invasive ventilation and neuromuscular. Retrospective, prospective, controlled, and non-controlled studies were included. Abstracts or clinical cases were excluded.

### Introduction of daytime support

The VC is commonly used to assess respiratory muscle weakness. A VC less than 50% [8] of predicted normal or forced expiratory volume in 1 s less than 40–50% predicted along with clear symptoms of sleep hypoventilation, indicate need to introduce sleep NVS. Maximal inspiratory pressure or sniff nasal pressure less than 30–40 cm H_2_O can also be helpful [21–29]. A decrease in VC of 25% or more when going from sitting to supine indicates diaphragm weakness and explains respiratory orthopnea and need for sleep NVS [51]. Typical symptoms of hypoventilation include fatigue, dyspnea, morning headaches, and hypersomnolence. Corroborating signs can include PaCO_2_ greater than 45 mmHg, nocturnal oximetry saturation < 88% for 5 consecutive minutes [21], and end-tidal CO2 > 47 mmHg [52]. Thus, the indication to initiate sleep NVS is symptomatic hypoventilation in the presence of any indications of respiratory muscle dysfunction. Typically, with advancing disease and decreasing VC, sleep NVS users become dyspneic when NVS is discontinued in the morning and nasal NVS is extended NVS into daytime hours [45, 52–60]. While sleep NVS initially normalizes daytime blood gases, further muscle deterioration causes increasing daytime hypercapnia such that when O_2_ desaturation occurs below 95% dyspnea results in ever increasing use of NVS throughout daytime hours [57–61].

### Daytime support methods

#### Mouthpiece and nasal NVS

Mouthpiece NVS was first described for CNVS in 1953 to permit iron lung users, with little to no autonomous ability to breathe, to leave iron lungs during the day. Subsequently they often began to refuse to return to iron lungs for sleep so they used mouthpiece NVS for CNVS [62, 63]. Their ventilator settings were typically pressure control at 18 to, at times for obese patients, 35 or more cm H2O for daytime as well as sleep NVS. When volume cycling portable ventilators came onto the market in 1976, CNVS was typically delivered at preset volumes of 800 to 1500 ml. The patients took as much of the air as desired for each breath. Besides normalizing PaO_2_, PaCO_2_, and sleep quality, CNVS indisputably prolongs survival for patients with little or no autonomous ability to breathe, 257 such patients were described in 1993 [64].

**Fig. 1:**
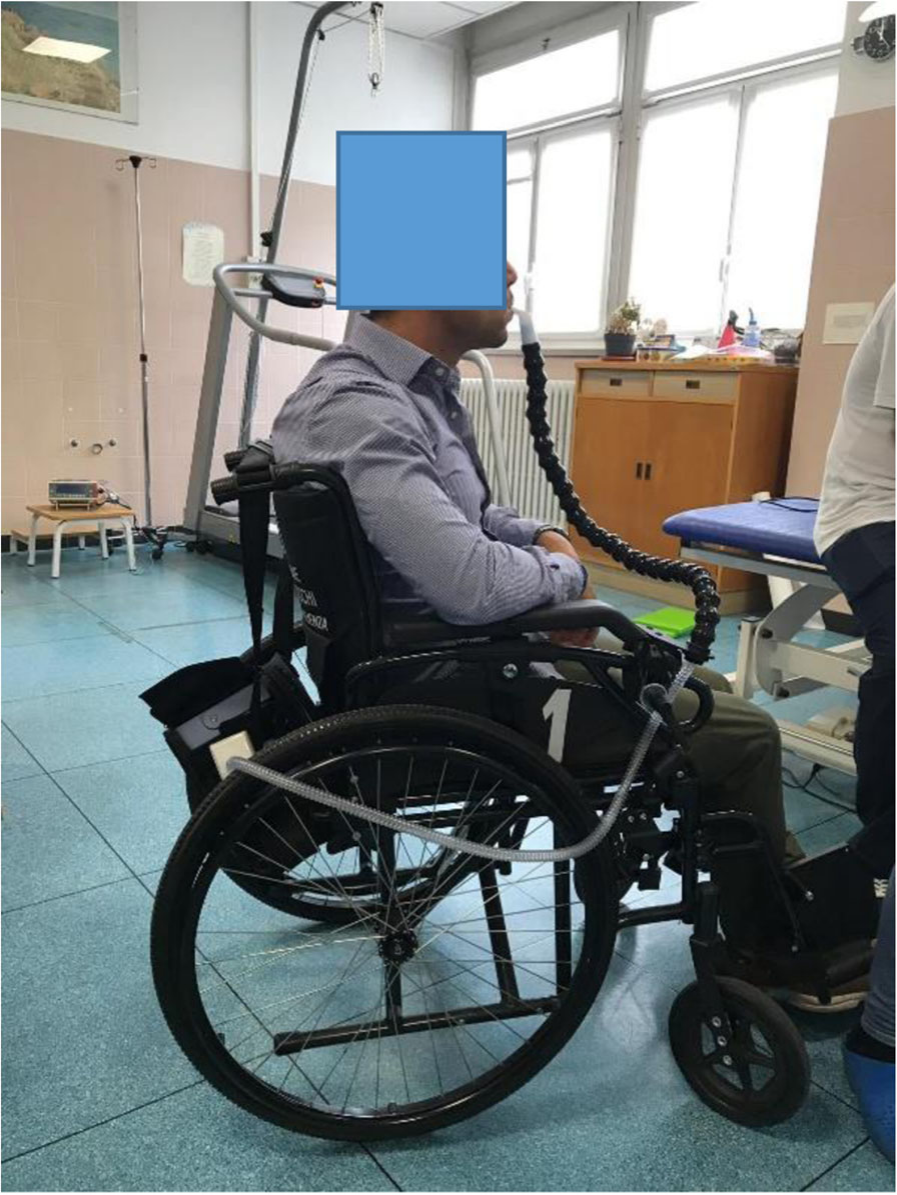
Mouthpiece ventilation

Mouthpiece NVS is provided via 15 or 22 mm angled, or straw-type, mouthpieces accessible to the mouth by being supported by a flexible support arm such that the patient can access it with the lips as needed (Fig. 1). Patients can trigger the breaths by creating a small negative pressure (sip and breathe) or placing the mouth on the mouthpiece (Kiss Trigger, Trilogy Ventilator, Respironics Inc., Murrysville Pa) [61]. Any mode can be used but volume preset ventilation with an active circuit permits active lung volume recruitment (“air-stacking”). This is the glottis retention of consecutively delivered volumes of air until a deep lung volume is attained. This maintains pulmonary compliance, increases cough flows and speech volume. A problem can be false alarms when using open circuits [61]. Several new portable ventilators have now a mouthpiece ventilation mode (“MPV mode”) that minimizes false alarms [65–68]. Although mouthpiece NVS facilitates speaking, eating, swallowing, and coughing, the patient can discontinue it as desired. There is no dyspnoea or transient oxyhemoglobin desaturations between grabbing the mouth piece for supported breaths when NVS settings are used [68]. For example, if normal minute ventilation is 6000 ml, this can be taken in by 12 breaths of 500 ml or 4 of 1500 ml. The latter affords more than 10 s for chewing and swallowing.

The 15 mm angled mouthpiece and straw-type mouthpiece are easy to grip [65, 68]. Khirani et al. [67] evaluated 209 patients, 30 using MPV modes, by questionnaire. These patients associated mouthpiece NVS with less dyspnoea and fatigue and greater facility in speaking and eating. In another study, Nicolini et al. [68] reported an improvement in clinical symptoms, blood gases, and in nocturnal ventilation, sleep related parameters and Health Related Quality of Live scores for kyphoscoliotic patients. While frequent or prolonged disconnections inducing apnoea/hypopnea or hypoventilation have been noted [63, 68], this only occurs when less than NVS settings are used.

Assist control pressure or volume preset ventilation modes with minimal back-up rates and extreme alarm settings are used to avoid nuisance alarms. The Kiss Trigger™ of the Trilogy™ ventilator permits 0 back-up rates [69].

While in the 1950s and 60s 15 mm angled mouthpiece NVS was used around-the-clock, once a lip cover phalange came onto the market in 1964 this secured the mouthpiece and permitted the delivery of air with little to no mouth leak during sleep (Fig. 1). This was the principal method of sleep NVS until Bach, Alba, Mosher, and Delaubier described nasal NVS in 1987. Today, 3 out of 4 patients prefer nasal over oral interfaces for sleep NVS [70, 71]. Recently, over 700 CNVS dependent muscular dystrophy and amyotrophic lateral sclerosis patients were reported using daytime mouthpiece NVS [72]. However, for those for whom lips are too weak or neck movement inadequate to grab a mouthpiece, daytime nasal NVS has always been preferred over resort to tracheotomy [71].

Skin breakdowns typically only occur when a single nasal interface is used around-the-clock [73, 74]. Daytime NVS can cause airway and mouth dryness that can negatively impact on quality of life and cause airway congestion and possibly inadequate gas-exchange and atelectasis [53]. With typical daytime mouthpiece NVS, however, there is no interface intolerance, difficulty speaking, expectorating or eating, facial deformities, or claustrophobia [16]. While skin discomfort, eye irritation, and other difficulties have been associated with the use of nasal and oronasal interfaces for sleep, none of this is relevant for daytime mouthpiece NVS users or nasal NVS users who use nasal prongs for daytime NVS and daytime mouthpiece or nasal prong NVS users.

#### Interfaces

Currently, there are many interfaces commercially available [74, 75] . They come in a variety of styles and sized i.e. nasal, oro-nasal, full-face, mouthpiece or orophylange. Each has advantages and disadvantages.

- Mouthpiece: Straw-type or 15 mm angled mouthpieces are grabbed with the lips and teeth [76]. No pressure over the bridge of the nose or need for headgear. It permits use on demand and active lung volume recruitment (air stacking). There are no downsides for daytime use other than mouth and airway dryness. The most commonly used mouthpiece is the 15 mm angled mouthpiece from Respironics Inc. (Murrysville, Pa) [16, 61, 76].
- .Nasal interface (mask): Although triangular nasal interfaces that cover only the nose are used for sleep, nasal prongs inserted into the nostrils, pillows, nose tip covering interfaces are greatly preferred for daytime support although they may cause nostril dryness. These avoid claustrophobia, permit eating and speech and mouth leaks are not a problem during daytime use. Triangular interfaces cause nasal bridge pressure.
- Oronasal interface (mask): These cover both nose and mouth. Mouth and nasal leaks are greatly reduced. They requires less patient cooperation but interfere with speech, eating, and expectoration. They increase risk of rebreathing and cannot be used during meals.
- Full-face interface (mask): These cover mouth, nose, and eyes. They can be useful for sleep but expose patients to eye redness and dryness; they interfere with speech, eating, and expectoration and can not be used for ongoing daytime NVS [75, 77, 78].
- Helmet: A transparent air-tight helmet rather than orofacial interface. These permit speech but the increased dead space and possibility of axillary skin injury prevent its ongoing daytime use [74, 75].

### Body ventilators

#### The intermittent abdominal pressure ventilator

**Fig. 2:**
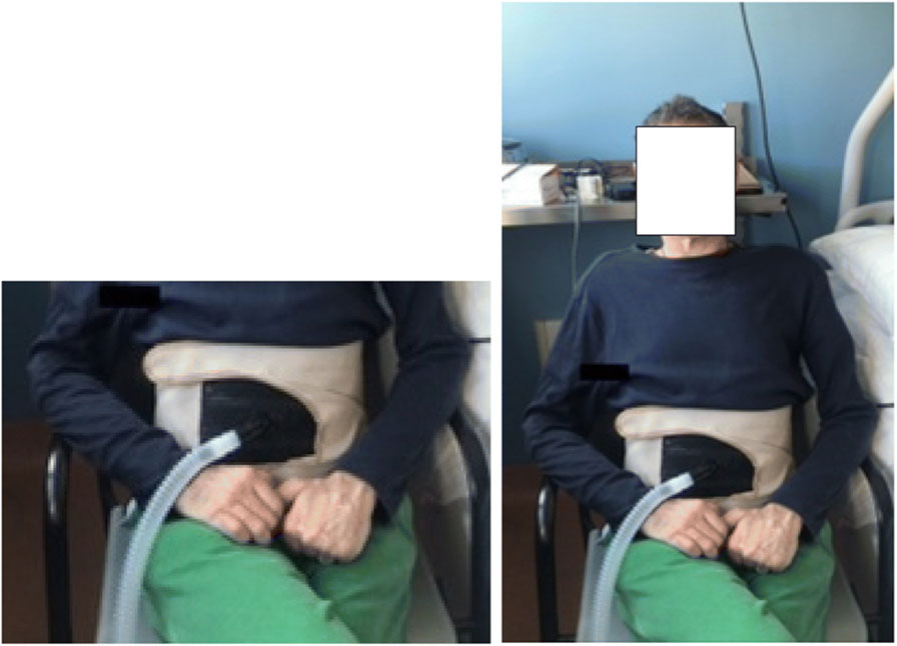
Intermittent abdominal pressure ventilation by PBelt

In 1938 the intermittent abdominal pressure ventilation,or “Pneumobelt, was described by C. J. McSweeney as the Bragg-Paul Pulsator. It was used by 34 patients with acute diphtheritic respiratory muscle paralysis. In the 1940s, it was improved by Dr. Alvin Barach and his engineer William Smith. After 1946, it became widely used for daytime non-invasive ventilatory support [79–84]. It consists of a corset with an elastic inflatable bladder that fits over the abdomen. The bladder is connected by a hose to a ventilator that deliver up to 2.5 l of air to the bladder and, thereby, to the abdominal wall [82, 83]. This elevates the diaphragm to cause expiration below the functional residual capacity. When the bladder deflates, the diaphragm descends by gravity and inspiration occurs passively. Typically, 300 to 1,000 ml of air are delivered [81]. It went off the market as tracheotomies became the convention but is back on the market today (Dima Inc., Italy). New models avoid clothing catching on the corset buckles and are more comfortable. They are now lightweight, comfortable, easy to done and fit and employ velcro for fastening (Fig. 2) [55]. Nevertheless, mouthpiece NVS users generally prefer mouthpiece NVS because of the greater inspiratory volumes that can be taken in.

Limitations include the fact that its efficiency depends upon the surface area of the chest and the abdomen covered. Since the increase of lung volume is generated by gravity, it is effective only in the sitting position [82], or at least at an angle of 30° or greater [44]. It is usually not sufficiently effective for very obese or severely scoliotic patients [79]. The current device on the market allows the user to set the following parameters: - PBelt (pressure inside the PBAir®); −Tinsp (real inspiratory time when the diaphragm descends); − Frequency (respiratory rate); and -Rise Time (time to reach the PBelt). Thus, “Pneumobelt” use is inconspicuous, comfortable, simple to do and use and leaves the face/mouth/nose, and neck free. It is portable and easily installed onto a wheelchair. It facilitates speech, belching to pass flatus and abdominal compression to increase cough flows to help clear secretions. Patients learn to close the glottis just prior to an abdominal compression to increase cough flows. It is also useful for constipation [82]. It also leaves the field of vision free and allows the patient a more normal sense of smell by eliminating facial and invasive interfaces that may be colonized by pathogenic bacteria. Thus, it favors oral nutrition and digestion [82]. In rare cases it is used for sleep with patients sitting [83]. Disadvantages can be food regurgitation during meals (rarely), catching clothing on straps and Velcro closures, redness of the bony prominences, and impossibility to shower or to bath when using it [83, 84]. Furthermore, regular follow up is important because it can become less effective with time [83, 84]. Its use has also recently been reported for patients with cervical myelopathies [55]. For all these reasons, the “Pneumobelt” is again becoming an important alternative for the patients requiring daytime support.

### Negative-pressure ventilation

Negative pressure ventilation was widely used for both sleep and daytime ventilatory support as iron lungs, pancho, pneumosuit, and pneumowrap ventilators before the advent of NVS in the 1950s. The chest cuirass is a rigid shell fitting over the anterior portion of the chest or over the chest and abdomen. The pneumowrap, poncho, pneumosuit ventilators consisted of a parka suspended over a rigid plastic or metal chest piece. The cuirass and wrap-style interfaces are powered by large negative pressure ventilators. While mouthpiece NVS facilitates speech, eating, maximizes coughing, and raising one’s voice, negative pressure body ventilators don’t.

Only the cuirass or chest shell ventilator can be used for daytime support for patients in wheelchairs [8–88]. The ventilator assumes part of the patient’s work of breathing and therefore respiratory muscles can rest but not so much as using NVS [89]. During sleep all negative pressure body ventilators cause severe obstructive apneas [90, 91].

The biphasic chest shell ventilator (Hayek Inc., England) can certainly provide daytime ventilatory assistance for patients without severe scoliosis, however it is unsightly, restrictive on mobility, and no more effective than “pneumobelts” at providing large tidal volumes for daytime ventilatory support. It has never been preferable to “pneumobelt” or mouthpiece NVS [82].

### Glossopharyngeal breathing (GPB)

Patients with at least some bulbar muscle function can often learn to glossopharyngeal breath for ventilator free breathing as well as for “auto” air stacking. This involves the tongue pistoning boluses of air past the glottis and into the lungs. It can be done with the mouth open or, in some cases, via the nose with the mouth closed [92–95]. It can improve speech volume and cough efficacy [95]. The technique can allow increased periods off of NVS or even all day autonomous ability for adept patients despite having little to no VC [96–99].

### Mechanical insufflation-exsufflation

Unfortunately, with the paradigm shift to invasive ventilatory support in the late 1960s, the Cof-flator, the first mechanical in-exsufflation device, went off the market as did the “Pneumobelt” 15 years later. There had been no major publications on it until 1991. However, like the “Pneumobelt”, mechanical in-exsufflation continues to be a practical, convenient, comfortable, and effective method to greatly increase cough flows to expel airway secretions and prevent pneumonia and respiratory failure and to permit extubation of ventilator unweanable patients to CNVS so that tracheotomy could be avoided indefinitely. Neither NIV and NVS should ever be considered in without considering mechanical in-exsufflation. In fact, publications reporting that NIV fails and tracheotomy is needed are simply failures to use NVS settings and, most importantly, failures to use mechanical in-exsufflation at the effective settings of 40 to 70 cm H_2_O via both noninvasive as well as invasive interfaces [100–102].

### Training

It is recommended that staff applying NVS receive appropriate training. Both specific knowledge and clinical experience are necessary. The training needs to be for health care professionals including physicians, nurses and physio/respiratory therapists, and most importantly, for the patient and family [103]. The time required for education and training in NIV/NVS and mechanical in-exsufflation is variable. Some authors have suggested that an initial session of two hours three times a month may provide adequate background [104]. With staff experience, sessions can then be distanced in time. Quantifying the experience and skill of a unit’s staff is challenging because individuals differ considerably and personnel changes can have important effects [105]. Optimal noninvasive management for daytime, as well as sleep support, requires all team members to be experienced. They must also be skilled in selecting appropriate devices and interfaces, fitting to optimize comfort, and adjusting the ventilator to efficiently alleviate respiratory distress and provide optimal settings. Nurses and physiotherapists need to be knowledgeable in regards to monitoring all stages of this in order to better train the patient and family.

## Conclusions

Daytime NVS is crucial for patients with advanced ventilatory pump failure to avoid acute on chronic respiratory failure and invasive airway tubes. Precise criteria for daytime NIV/NVS initiation are totally unnecessary since patients extend nocturnal use into daytime hours on their own. Interfaces and access to mechanical in-exsufflation play key roles for NVS success. Glossopharyngeal breathing can also be a very effective means of daytime ventilatory support. Body ventilators may become a more prominent feature of daytime support with time. Further research in this emerging field is strongly warranted in order to further improve the quality of breathing and living for these fragile patients.

## Data Availability

Not available.

## Abbreviations

NIV: Noninvasive Ventilation
CNVS: Continuous Noninvasive Ventilatory Support
NVS: Non-invasive Ventilatory Support
VC: Vital Capacity
MPV: Mouthpiece Ventilation
